# Platelet Recovery and Coexisting Conditions in Pediatric Immune Thrombocytopenia: Insights from a Tertiary Care Study

**DOI:** 10.3390/children12111482

**Published:** 2025-11-03

**Authors:** Cristina Elena Singer, Mocanu Andreea Gabriela, Sîrbuleț Carmen, Anca Enescu, Simina Gaman, Renata Maria Varut, Dop Dalia, Maria Elena Veronica, Ștefănița Bianca Vintilescu, Nina Ionovici, Pluta Ion Dorin, Arsenie Cristian-Cosmin, Cristina Popescu

**Affiliations:** 1 Department of Mother and Baby, University of Medicine and Pharmacy of Craiova, 200349 Craiova, Romania; cristina.singer@umfcv.ro (C.E.S.); dalia.dop@umfcv.ro (D.D.); veronica.maria@umfcv.ro (M.E.V.); bianca.vintilescu@umfcv.ro (Ș.B.V.); nina.ionovici@umfcv.ro (N.I.); 2 Department of Pharmaceutical Technology, Faculty of Pharmacy, University of Medicine and Pharmacy of Craiova, 2 Petru Rares Street, 200349 Craiova, Romania; gabriela.mocanu@umfcv.ro; 3 Department of Anatomy, University of Medicine and Pharmacy of Craiova, Discipline of Anatomy, 200349 Craiova, Romania; carmen.sirbulet@umfcv.ro (S.C.); anca.enescu@umfcv.ro (A.E.); arsenie_cristian@yahoo.com (A.C.-C.); cristina.popescu@umfcv.ro (C.P.); 4 Department I, Faculty of Dental Medicine, University of Medicine and Pharmacy of Craiova, 200349 Craiova, Romania; 5 Research Methodology Department, Faculty of Pharmacy, University of Medicine and Pharmacy of Craiova, 200349 Craiova, Romania; renata.varut@umfcv.ro; 6 Faculty of Medical and Behavioral Sciences, Constantin Brâncuși University of Târgu Jiu, 210185 Târgu Jiu, Romania; dorin.pluta@e-ucb.ro

**Keywords:** immune thrombocytopenia, hematologic disorder, platelet recovery, observational study, petechiae and ecchymoses

## Abstract

Background/Objectives: Immune thrombocytopenia (ITP) is a rare but significant hematologic disorder in children. While most pediatric ITP cases resolve spontaneously, some require intervention, and treatment responses with platelet recovery patterns can vary widely. We conducted a retrospective, three-year observational cohort study including 100 children (aged 5 months–17 years) diagnosed with ITP at a single tertiary center (January 2022–December 2024). The primary objective of this study was to characterize the clinical presentation, coexisting conditions, and short-term platelet response to first-line therapy in hospitalized children with newly diagnosed ITP. Methods: Clinical data (demographics, presentation, and laboratory results) were collected at admission and throughout hospitalization, and outcomes were assessed at discharge. Results: Our analysis showed a balanced urban–rural distribution overall; however, sex distribution differed by residence, with a slight female predominance in rural areas and an overwhelming male predominance in urban areas. Over 40% of patients were under 5 years old. Platelet counts at presentation ranged from 3 × 10^9^/L to 30 × 10^9^/L, yet nearly all children showed substantial platelet count recovery by hospital discharge under first-line therapy. Most patients had additional minor clinical findings at admission, but these were considered concurrent rather than causative factors, in accordance with current hematology guidelines. These conditions were considered coexisting clinical conditions or concurrent findings rather than definitive secondary causes of ITP, in accordance with established hematological guidelines. These findings reflect only short-term, in-hospital outcomes and may not be generalizable to all children with ITP, particularly milder or outpatient cases. Conclusions: Our three-year cohort study underscores that pediatric ITP often presents in very young children with concurrent conditions, but standard first-line treatment leads to robust platelet recovery by discharge. These findings highlight the importance of comprehensive initial evaluation and supportive care in managing pediatric ITP.

## 1. Introduction

Immune thrombocytopenia (ITP) is an immune-mediated hematologic disorder characterized by a transient or persistent decrease in platelet count and an associated risk of bleeding, depending on the degree of thrombocytopenia [1]. Although ITP is rare, it is the most common cause of acquired thrombocytopenia in childhood, with an incidence of 2 to 5 per 100,000 children annually [2]. Diagnostic criteria include a platelet count of less than 100 × 10^9^/L and exclusion of other causes of thrombocytopenia. According to the International Working Group, ITP is classified by duration as newly diagnosed (up to 3 months), persistent (3–12 months), and chronic (>12 months), and by etiology as primary (idiopathic) or secondary (associated with infections or autoimmune diseases) [3]. Consistent with this, previous studies have noted that pediatric ITP frequently coincides with conditions such as hypocalcemia, herpetic stomatitis, and rhinosinusitis/adenoiditis [4].

The pathogenesis of ITP involves two key mechanisms: reduced platelet production in the bone marrow and increased platelet destruction due to antiplatelet autoantibodies, the latter being generally predominant [5]. Historically, the disease was attributed primarily to autoantibodies targeting platelet surface glycoproteins, resulting in premature clearance of platelets by the reticuloendothelial system [3]. However, recent research has revealed that impaired megakaryocyte function, defective platelet production, and immune dysregulation—including an imbalance between pro-inflammatory and regulatory T cells, also contribute to disease persistence and severity [6]. These multifactorial mechanisms underscore the complexity of ITP and the need for further research into the interplay between immune-mediated platelet destruction and impaired thrombopoiesis. Moreover, pediatric ITP frequently coexists with inflammatory or metabolic disturbances such as herpetic stomatitis, adenoiditis, and transient hypocalcemia. These conditions may contribute to immune activation and platelet destruction through shared pathogenetic pathways. Viral or bacterial infections of the upper airway and oral mucosa can trigger cytokine release (e.g., IL-6, TNF-α) that enhances macrophage-mediated platelet clearance. Similarly, calcium plays a central role in platelet activation and aggregation; transient hypocalcemia may reflect increased cellular consumption during immune activation or altered vitamin D metabolism, indirectly influencing megakaryocyte function and platelet production. Recent studies suggest that these metabolic or infectious co-triggers may modulate disease severity and therapeutic responsiveness in children with ITP [7,8].

Diagnosing ITP remains challenging, as it is essentially a diagnosis of exclusion. Clinicians must rule out other causes of thrombocytopenia, including bone marrow failure syndromes, infections, and malignancies, before confirming ITP. The absence of a definitive diagnostic test necessitates reliance on clinical judgment and thorough patient history, further complicating early detection and prognostication. This uncertainty is compounded by the heterogeneous nature of ITP, with some children experiencing spontaneous remission while others progress to chronic disease requiring prolonged treatment [9].

The clinical and prognostic spectrum of pediatric ITP is highly variable. While most cases are self-limiting, a significant proportion progress to chronic forms associated with recurrent bleeding and the need for long-term management. First-line therapies such as intravenous immunoglobulin (IVIG) and corticosteroids yield variable responses, emphasizing the need for reliable predictors of outcome [10]. Understanding epidemiological trends, clinical presentations, and recovery patterns in pediatric ITP is crucial for optimizing treatment and improving quality of life for affected children and families [11,12].

Treatment strategies for ITP are versatile and may include observation, IVIG, corticosteroids, thrombopoietin receptor agonists (TPO-RAs), or even splenectomy [13,14]. Splenectomy, historically regarded as a curative approach for refractory ITP, is now reserved for chronic or treatment-resistant pediatric cases due to the risk of infection and the availability of thrombopoietin receptor agonists. However, clinical practice remains heterogeneous due to the diversity of concepts, definitions, and therapeutic approaches [15]. Considering these challenges, our study was designed to address existing knowledge gaps by providing a comprehensive observational analysis of pediatric ITP over a three-year period. The primary aim was to determine the key factors influencing platelet recovery and to elucidate the epidemiological and clinical characteristics associated with both acute and chronic forms of ITP in children.

## 2. Materials and Methods

### 2.1. Study Design

This study was designed as a retrospective observational cohort investigation of pediatric patients diagnosed with ITP over a three-year period (January 2022 to December 2024). A total of 100 children (aged 5 months to 17 years) who met the diagnostic criteria for ITP were included. The diagnosis of ITP was established according to the criteria outlined by the International Working Group (IWG) and the American Society of Hematology (ASH) guidelines [1,2]. In all patients, the diagnosis of ITP was established based on a careful exclusion of alternative causes of thrombocytopenia, in accordance with the ASH 2019 guidelines and IWG consensus criteria [1,2]. Clinical history, physical examination, and extensive laboratory investigations (complete blood count with peripheral smear, infection screening, autoimmune markers, renal and hepatic function, electrolytes, including calcium levels) were performed for each case. No clinical features or laboratory abnormalities suggestive of congenital bone marrow failure syndromes, immune deficiencies, malignancies, metabolic disorders (including Fahr or Stormorken syndromes), or 22q11.2 deletion syndrome were identified. Brain imaging was performed when indicated, and no intracranial calcifications were detected. Specifically, ITP was defined as a platelet count <100 × 10^9^/L in the absence of other causes or disorders that may be associated with thrombocytopenia. Patients were eligible if they had primary (idiopathic) ITP or secondary ITP associated with an underlying condition (e.g., infection), as long as immune-mediated thrombocytopenia was the presumptive mechanism. Key exclusion criteria were any alternative causes of thrombocytopenia, such as bone marrow failure syndromes, hematologic malignancies, or drug-induced thrombocytopenia. Only newly diagnosed ITP cases were included in this cohort. In accordance with international classification guidelines, ITP is categorized as newly diagnosed (<3 months), persistent (3–12 months), and chronic (>12 months) [1]. Given the absence of follow-up in our study, we were only able to include cases classified as newly diagnosed ITP at the time of admission. Patients with any history of prior ITP episodes or relapsing/chronic ITP were excluded to maintain a homogenous population of incident cases. This study included only children who were hospitalized in the Pediatric Hematology Department of the Emergency County Clinical Hospital of Craiova. Patients who were evaluated in the Emergency Department but not admitted were excluded from the analysis. This inclusion criterion was applied to ensure complete clinical, laboratory, and treatment data for all cases. For the purposes of this study, urban and rural residences were classified according to the Romanian National Institute of Statistics administrative categories, based on the patient’s official home address as recorded in hospital medical records. “Urban” corresponded to residence within an incorporated city or town, and “rural” referred to residence within communes or villages.

All enrolled patients underwent a comprehensive evaluation at admission to characterize their hematologic status and to screen for possible precipitating factors or comorbid conditions. The following laboratory and clinical investigations were performed systematically for each patient:

➢ Hematologic tests: Complete blood count with differential, including serial platelet count measurements at presentation and throughout the hospital stay to monitor thrombocytopenia severity and recovery.
➢ Serum calcium: Measured to detect any calcium imbalance (e.g., hypocalcemia), given that metabolic disturbances were of interest in this cohort.
➢ Serum iron: Evaluated to assess iron levels and identify any coexistent iron deficiency or anemia.
➢ Liver enzyme panel: Assessment of hepatic function via aspartate aminotransferase, alanine aminotransferase, and gamma-glutamyl transferase.
➢ Renal function tests: blood urea, creatinine, and uric acid levels were measured to evaluate kidney function and to rule out any renal impairment or tumor lysis–like syndrome that could contribute to laboratory anomalies.
➢ Serum electrolyte panel: Measurement of key electrolytes (such as sodium, potassium, chloride, and bicarbonate) to detect any electrolyte imbalances.
➢ Urine analysis: A complete urinalysis was performed to screen for hematuria, proteinuria, or signs of infection that might be present alongside ITP.
➢ Fundoscopic examination: An ophthalmologic exam of the ocular fundus (both eyes) was conducted at admission to check for retinal hemorrhages or other abnormalities, as severe thrombocytopenia can predispose to retinal bleeding.
➢ Chest radiograph: A chest X-ray was obtained to identify any pulmonary infiltrates or mediastinal abnormalities, and to evaluate for signs of infection or lymphadenopathy that could suggest a secondary cause of thrombocytopenia.
➢ Viral panel: Screening for viral infections known to trigger secondary ITP was done by testing for serological evidence of the TORCH panel (Toxoplasma, Rubella, Cytomegalovirus, Herpes simplex virus types 1 and 2 (HSV-1, HSV-2)), Epstein–Barr virus (EBV).
➢ Infectious disease markers: Additional serologic tests were performed for hepatitis C virus (anti-HCV antibodies), hepatitis B (hepatitis B surface antigen, HBsAg), and human immunodeficiency virus (HIV-1/2 antibodies) to rule out these chronic infections as contributors to thrombocytopenia.
➢ Lactate dehydrogenase (LDH): Serum LDH levels were measured as a non-specific marker of cell turnover or hemolysis, aiding in the assessment of disease severity and in distinguishing ITP from other hemolytic processes.

All laboratory analyses were conducted in the hospital’s certified clinical laboratory using standard methods. Blood samples were collected at admission (prior to any treatment), then daily during hospitalization, and at discharge to monitor platelet recovery. Patients with marked thrombocytopenia received discharge instructions for home monitoring and were advised to attend outpatient follow-up visits according to standard clinical care; however, no data were systematically collected after discharge, and all study outcomes were assessed at the time of hospital discharge. All patients received IVIG, and only those presenting with significant bleeding or at high risk for life-threatening hemorrhage also received transfusions with platelet concentrates. Cases with non-life-threatening hemorrhagic symptoms received short-term corticosteroid therapy (prednisone) for 7 days. Bone marrow aspiration was performed only in selected cases with atypical clinical or laboratory findings or when diagnostic uncertainty remained after initial evaluation. Additional investigations included coagulation panels, direct Coombs tests, D-dimers, antinuclear antibodies (ANA), serum immunoglobulins, and abdominal imaging (ultrasound or CT) to exclude splenomegaly or other organ involvement. In selected cases, endocrinology consultations were solicited for further evaluation of systemic coexisting clinical conditions. CBCs were performed using an automated hematology analyzer (Sysmex XN-series), while serum biochemistry (including electrolytes, calcium, LDH, and liver/renal function tests) was measured on a high-throughput analyzer (Roche Cobas). All assays followed strict quality control procedures with daily internal controls and participation in external proficiency testing under ISO 15189 accreditation [16].

The study was conducted in accordance with the principles of the Declaration of Helsinki and approved by the Institutional Ethics Committee of the Emergency County Clinical Hospital of Craiova, Romania (Approval No. 12.232/2025). Written informed consent was obtained from the parents or legal guardians of all participating children.

At our tertiary care center, children presenting with newly diagnosed ITP are routinely admitted for diagnostic evaluation and initial therapy when platelet counts fall below 30 × 10^9^/L or when bleeding manifestations are evident. Management follows the Romanian national pediatric hematology protocols, which are harmonized with international (ASH 2019) guidelines [1,2]. IVIG represents the standard first-line treatment, while short-course corticosteroids (prednisone) are administered when IVIG is contraindicated or not readily available. Platelet transfusions are reserved for patients with active or life-threatening bleeding and platelet counts below 10 × 10^9^/L. Outpatient observation is generally reserved for milder cases with platelet counts above 30 × 10^9^/L and no bleeding symptoms.

### 2.2. Statistical Analysis

Data were analyzed using R (version 4.0.3; The R Foundation for Statistical Computing, Vienna, Austria). Continuous variables are presented as medians with interquartile ranges (or means ± standard deviation), and categorical variables as counts and percentages. We used the chi-square test (or Fisher’s exact test when appropriate) to compare categorical variables, and Student’s *t*-test or Mann–Whitney U-test to compare continuous variables between groups. A *p*-value < 0.05 was considered statistically significant for all analyses.

## 3. Results

### 3.1. Age Prevalence

**Figure 1:**
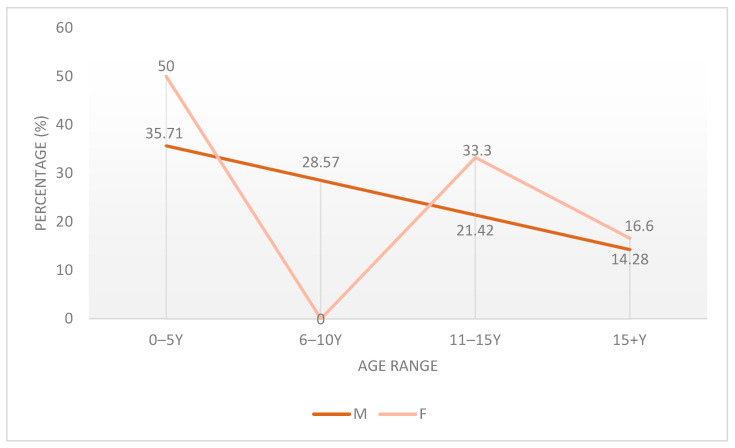
Percent of Male/Female patients in different age ranges.

In this study, patient ages ranged from 5 months to 17 years, with a clear male predominance (70% male, 30% female). Most patients were in the youngest age group (0–5 years), followed by the 11–15 years category. Notably, the 6–10 years group consisted entirely of male patients. Among female patients, most were aged 0–5 or 11–15 years, while no females were present in the 6–10 years group. This age and sex distribution is detailed in Table 1 and Figure 1.

### 3.2. Patient’s Background

As for the patients’ background, 45% of the patients were from rural areas and 55% from urban areas. While the overall rural–urban distribution appeared balanced, sex-based analysis revealed a statistically significant difference (*p* = 0.01): the rural subgroup showed a slight female predominance (55.5% female), whereas the urban subgroup was overwhelmingly male (90.9% male).

In the rural area, where 55.5% of patients were female, 60% of them were between 0 to 5 years old, and 40% were between 11 to 15 years. As for the males, as high as 40% from the rural background were included in the 6–10 years range, with 25% in both 0–5 and 15+ years, while none of the male patients from the rural background were from 11 to 15 years. In the urban area, male prevalence was 90.9%. Among these male patients, 40% were between 5 months and 5 years of age, 20% were aged 6–10 years, 30% were between 11 and 15 years, and only 10% were older than 15 years.

### 3.3. Platelet Count

One of the most clinically relevant parameters in thrombocytopenia is the platelet count at hospital admission. The majority of children (35%) presented with severe thrombocytopenia, having platelet counts between 0 and 5000/µL. An additional 20% had platelet levels in the range of 5000 to 10,000/µL, while 15% exhibited counts between 10,000 and 15,000/µL. Moderate thrombocytopenia, defined as platelet counts from 15,000 to 30,000/µL, was observed in another 20% of cases. Only a small proportion (10%) of patients presented with platelet counts exceeding 30,000/µL. These findings highlight that the vast majority of patients (90%) were admitted with platelet counts below 30,000/µL, underscoring the severity of thrombocytopenia in this hospitalized cohort.

**Figure 2:**
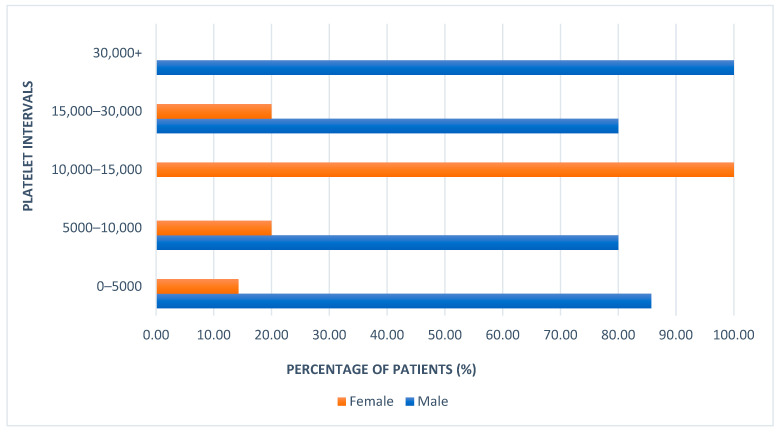
Number of platelets in male/female patients at admission.

Among the patients with platelet counts below 5000, 85.71% were male and 14.28% were female (Figure 2). In addition, male patients represented 100% of the cases in two categories: 5000–10,000 and more than 30,000 platelets. At the same time, the remaining female patients had platelet counts between 10,000–15,000, where their representation was 100%. As we can observe, male patients were predominant in both the lowest and highest platelet count categories at hospital admission, while female patients were mainly clustered in the 10,000–15,000 range.

The difference in platelet counts between admission and discharge was also evaluated. The largest increases in platelet count were observed in patients who had fewer than 5000 platelets at admission. Additionally, higher platelet counts at discharge were noted among male patients, with the highest recorded value being 475,000, compared to a maximum of 300,000 in female patients.

For patients with initial counts below 5000, the post-treatment arithmetic mean was 277,428.57. In the 5000–10,000 category, the mean was 242,500; in the 10,000–15,000 group, it was 178,666; for the 15,000–30,000 group, the mean reached 255,000; and among those with more than 30,000 platelets at admission, the mean was 222,000. The lowest post-treatment count was observed in the category predominantly composed of female patients, while the highest was seen in the male-dominated group, where female representation was 14.28%.

Platelet counts increased significantly from admission to discharge (median rise from 22 × 10^9^/L [IQR 12–45] to 152 × 10^9^/L [IQR 110–200]; *p* < 0.001). This marked improvement was predominantly attributable to the therapeutic effect of first-line treatment, particularly IVIG and, in selected cases, short-course corticosteroids. The rapid platelet rise observed over a few days is consistent with the known response kinetics to these interventions rather than spontaneous remission. This increase was observed in both sexes, with no significant difference between boys and girls at baseline or after treatment (*p* > 0.4). Stratification by platelet count categories showed no association between sex or age and severity at presentation (*p* > 0.5), although those with <5 × 10^9^/L had the largest absolute platelet increases.

**Figure 3:**
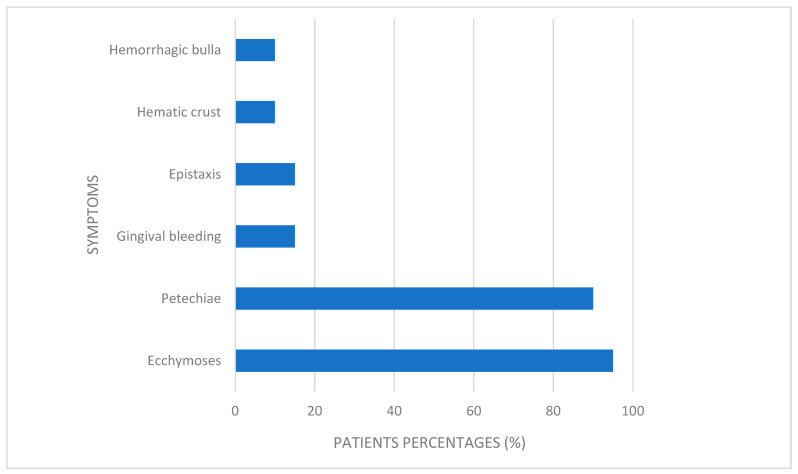
Symptom prevalence.

Figure 3 illustrates the increase in the arithmetic mean platelet count after treatment across each admission platelet count category. The arithmetic mean of platelet counts before and after treatment was calculated separately for male and female patients, with higher values observed in male patients.

### 3.4. Symptom Classification

Regarding symptoms, the most common was ecchymosis, affecting 95% of patients, with no sex difference (*p* > 0.05). All female patients presented with ecchymoses, while the prevalence among male patients was 92.82%. The second most common symptom was petechiae, observed in 90% of the cohort—83.3% of female patients and 92.85% of male patients exhibited this manifestation, showing a statistically significant difference between sexes (*p* = 0.04). Less common symptoms included gingival bleeding and epistaxis, each present in 15% of cases, as well as hematinic crust and hemorrhagic bullae, each reported in 10% of patients, showed no sex-based differences (*p* > 0.5) (Figure 3).

**Figure 4:**
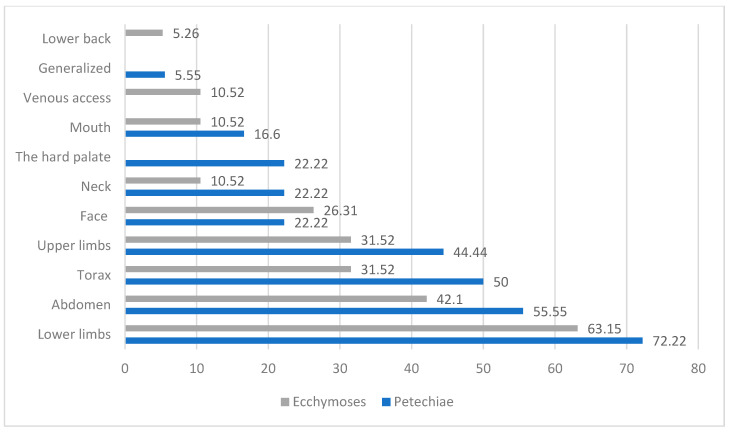
Localization of the most prevalent symptoms (with percentage values indicated for each site of bleeding).

**Figure 5:**
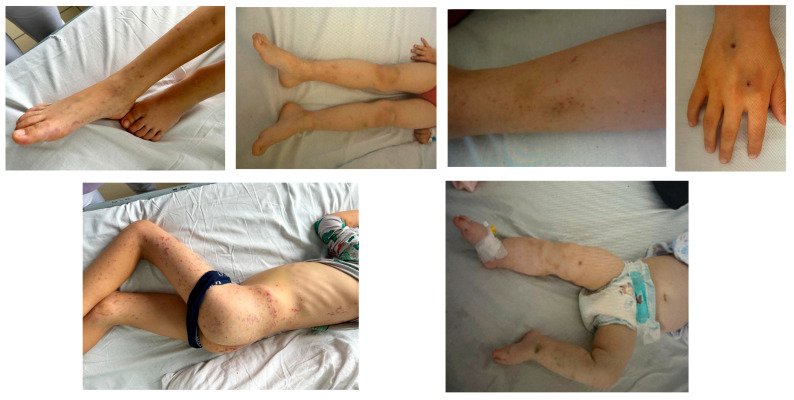
Clinical presentation of petechiae and ecchymoses in selected patients. All images are original and were obtained from patients included in our study, with prior written informed consent from parents or legal guardians.

Regarding the localization of ecchymoses (Figure 4 and Figure 5), the most frequent sites were the lower limbs (63.15%), abdomen (41.1%), and the thorax and upper limbs (31.52%).

Table 2 presents the localization of petechiae and ecchymoses, observing the distribution across different anatomical sites. Furthermore, it shows the percentage of sex prevalence for each localization, providing insight into potential sex-based differences in symptom presentation. As we can see, a very high number of petechiae localized on lower limbs, the abdomen, and upper limbs and generalized petechiae were found in male patients.

Coexisting clinical conditions were present in 85% of patients. The most frequent was hypocalcemia (45%), followed by infections (herpetic stomatitis, rhinosinusitis) and nutritional imbalances. However, no significant sex differences were found in the prevalence of comorbid conditions (*p* > 0.5).

**Figure 6:**
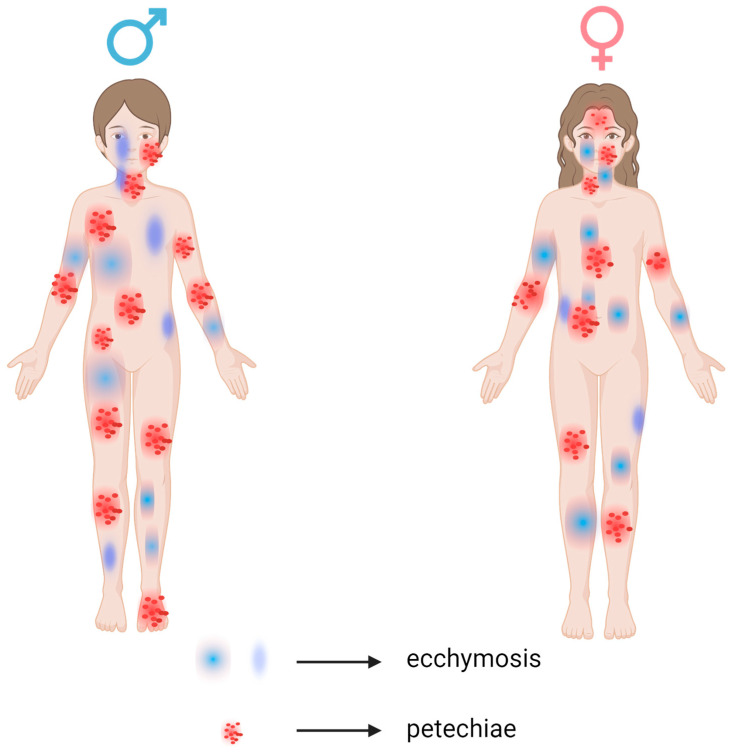
Sex-Based Anatomical Distribution of Petechiae and Ecchymoses in Patients with Immune Thrombocytopenia.

Meanwhile, female patients had a greater occurrence of petechiae localized to the face and mouth (Figure 6).

### 3.5. Treatment

Treatment was initiated according to institutional and national pediatric hematology protocols, aligned with international (ASH 2019) recommendations [1,2]. All treatment decisions were made by the attending pediatric hematologists of the Pediatric Hematology Department, in accordance with standardized hospital protocols and after multidisciplinary review when required. First-line therapy for all patients consisted of intravenous immunoglobulin (IVIG), consistent with current international guidelines for newly diagnosed ITP in children. In Romanian tertiary pediatric hematology centers, IVIG represents the preferred first-line therapy for newly diagnosed ITP, particularly in hospitalized children with severe thrombocytopenia or mucocutaneous bleeding, owing to its rapid onset of action and established safety profile. Short-course corticosteroid therapy (oral prednisone, 5–7 days) is used in selected cases, including when IVIG is contraindicated, unavailable, or in children with milder, non-life-threatening bleeding. This approach aligns with the Romanian national pediatric hematology guidelines, which are harmonized with international recommendations but prioritize IVIG in inpatient settings to achieve faster platelet recovery. Platelet transfusions were not administered universally, but were reserved for selected cases presenting with active, clinically significant bleeding or at high risk for life-threatening hemorrhage, in accordance with institutional protocols and current international recommendations. So, platelet transfusions were given selectively to 22 (from the total of 55) patients with active bleeding or risk of and platelet counts <10 × 10^9^/L, in accordance with pediatric hematology protocols. Children with low platelet counts but without bleeding symptoms were managed conservatively without prophylactic transfusion, while corticosteroid therapy (oral prednisone) was administered to 26 patients, particularly those with non-life-threatening hemorrhagic symptoms or when IVIG was contraindicated or unavailable. Among supportive medications, desloratadine was prescribed in 40% of cases, primarily for allergic symptom management. These treatments were given alongside standard ITP therapy to manage each patient’s coexisting clinical conditions. Gluconate was administered to 30% of patients, addressing the frequently observed hypocalcemia. Gastrointestinal protection was ensured with omeprazole in 20% of patients, while rutoside (biflavonoid), a dietary supplement, was also used in 20% of cases to support nutritional needs. Cefoperazone–sulbactam and meropenem were each administered in 5% of cases, likely for treating bacterial infections. Similarly, acetaminophen and vitamin C were each prescribed to 5% of patients, serving antipyretic/analgesic and antioxidant roles, respectively. The use of these adjunctive medications reflected a combination of standardized hospital protocols and case-by-case clinical judgment (Table 3).

### 3.6. Diagnosis

The classification by diagnosis at hospital discharge is presented in Table 4. We reported the percentage of patients for each diagnostic category, along with the distribution by sex. The most common condition associated with ITP was mild/moderate hypocalcemia, present in 45% of patients (30% mild and 15% moderate hypocalcemia). Although the sex distribution within this subgroup was nearly equal, the total percentage of male patients with hypocalcemia was lower (64.28%) compared to female patients (66.6%) due to the higher overall number of male patients in the cohort.

Additionally, 20% of patients had herpetic stomatitis or rhinosinusitis/adenoiditis. These infections may indicate increased susceptibility or altered immune function in children with ITP. Regarding nutritional status, obesity was observed twice as frequently as underweight, with male patients predominating in the obesity group, while the underweight category showed an even sex distribution. There was no diagnostic category in which female patients predominated, except for acute pneumonia. In contrast, male patients accounted for 100% of cases involving herpetic stomatitis, rhinosinusitis/adenoiditis, epistaxis, serous otitis, hyponatremia, epilepsy, and maxillary sinusitis. Interestingly, all 15% of patients diagnosed with isolated ITP (without coexisting clinical conditions) were male. Although all cases of isolated ITP in our cohort occurred in boys, this observation may reflect sampling variability or selection bias inherent to a single-center, retrospective study. Therefore, these demographic findings should be interpreted with caution and require confirmation in larger, multicenter cohorts. All patients included in the study presented with isolated thrombocytopenia, with no cases of additional cytopenias at admission. Bone marrow aspiration was performed in 12 patients (12%) who had atypical clinical or laboratory features or diagnostic uncertainty; all bone marrow examinations revealed findings compatible with ITP (normal or increased megakaryocyte counts and no evidence of malignant or infiltrative disease). Furthermore, all patients were systematically evaluated for underlying immunodeficiencies, especially those with coexisting hypocalcemia, pneumonia, or recurrent infections. This assessment included detailed medical history, physical examination, and measurement of serum immunoglobulin levels (IgG, IgA, IgM), which were found to be within age-appropriate reference ranges in all cases. No patient exhibited features suggestive of inborn errors of immunity.

## 4. Discussion

### 4.1. Demographic Characteristics and Sex-Based Differences

Our pediatric ITP cohort exhibited distinct demographic patterns compared to adult cases. Whereas adult ITP predominantly affects young women [17,18], our findings showed a striking male predominance in children (only ~30% of patients were female). Notably, we observed that no female patients were included in the 6–10 years age group. Although this finding could reflect random variation within our cohort, it raises the possibility of sex-specific or age-dependent susceptibility patterns. Further large-scale epidemiological studies are needed to explore whether such distributional gaps are statistically and biologically meaningful.

Romania presents marked disparities between urban and rural areas in terms of socioeconomic status, healthcare access, and environmental exposures. Rural communities often face limitations such as reduced access to specialist care, delayed pediatric or hematology consultations, and lower rates of vaccination and infectious disease screening. In addition, socioeconomic challenges, including lower parental education levels, suboptimal nutrition, and limited health literacy, may influence both the presentation and management of hematologic disorders like ITP. These structural disparities may partially explain some of the patterns observed in our cohort, including differences in age distribution, sex ratio, or the prevalence of coexisting conditions between urban and rural children [19].

Our data revealed that over 40% of patients were under 5 years old, and a notable proportion originated from rural areas. The observed male predominance is consistent with findings from other large pediatric ITP studies. Epidemiological data from national registries have generally reported balanced sex distributions, but minor sex-based variations can occur, especially in single-center, retrospective cohorts. Such findings should therefore be interpreted with caution [20].

Epidemiological data from national registries have shown relatively balanced sex distributions, with slight variations across age groups. For example, Shaw et al. analyzed over 4000 cases of childhood ITP and reported a nearly equal male-to-female ratio. This suggests that minor sex-based variations may occur in individual cohorts, particularly those derived from single-center, retrospective data, and such differences should be interpreted with caution [20]. It is important to emphasize that this study reflects only the inpatient clinical course of children hospitalized with newly diagnosed ITP. Due to the absence of post-discharge data, we cannot draw conclusions regarding long-term outcomes, chronicity, or relapse. Moreover, as a hospital-based sample, our cohort may overrepresent more severe cases requiring admission, introducing a degree of selection bias. As such, our study did not include long-term follow-up data and was unable to classify cases according to the full international criteria (i.e., persistent and chronic ITP forms). We included children at the point of initial diagnosis and excluded patients with recurrent or previously treated ITP. This methodological decision limits our ability to evaluate disease duration or evolution beyond hospitalization. This epidemiological profile suggests that pediatric ITP may have unique risk factors and triggers. Indeed, environmental and socio-demographic influences have been reported to impact ITP incidence.

### 4.2. Clinical Presentation and the Prevalence of Coexisting Clinical Conditions in Pediatric ITP

Moreover, while it is often assumed that most cases of childhood ITP are idiopathic, in our cohort, only 15% of patients presented with isolated primary ITP [21]. The remaining 85% exhibited one or more coexisting clinical conditions at admission. However, consistent with current hematological guidelines, these findings were interpreted as coincidental or accompanying conditions rather than definitive secondary causes of ITP. This distinction is crucial, as true secondary ITP, attributable to well-defined triggers such as autoimmune diseases, persistent infections, or specific medications, remains relatively uncommon, particularly in young children. Our observations highlight that minor infections and metabolic disturbances frequently coexist with ITP in pediatric populations without necessarily implying an etiological relationship [22,23]. Collectively, these demographic and epidemiologic insights provide important context for interpreting our clinical findings. In terms of clinical presentation, bleeding manifestations in our patients were generally mild and involved primarily cutaneous or mucosal sites [24]. Serious hemorrhages were rare despite the profoundly low platelet counts at admission (platelet counts ranged from 3 × 10^9^/L up to 30 × 10^9^/L in our cohort). Notably, many children with platelet counts below 10 × 10^9^/L showed minimal or no bleeding symptoms. This reflects the well-recognized weak correlation between platelet count and bleeding severity in ITP [25]. None of our patients needed second-line interventions, highlighting the effectiveness of standard first-line therapy in this acute setting. Overall, our findings affirm that with appropriate first-line management, children with ITP usually experience swift and meaningful platelet recovery, consistent with outcomes reported in the literature. A systematic review further suggests that prompt IVIG treatment may reduce the likelihood of chronic ITP developing in pediatric patients [26,27,28,29,30,31]. In addition to IVIG, we frequently utilized prophylactic platelet transfusions, a practice supported by studies showing that platelet support can reduce bleeding risk in severe thrombocytopenia [32,33]. Our experience reinforces that some children can tolerate extremely low platelet counts without major bleeding, likely due to compensatory mechanisms. Therefore, clinical judgment—rather than an absolute platelet threshold—should guide management. The overarching goal in ITP therapy is to prevent significant hemorrhage, emphasizing that treatment is driven by bleeding risk more than by the platelet count itself [18]. All patients in our study were treated with first-line therapy, consisting of IVIG and adjunctive platelet transfusions as needed. The response was excellent: most patients achieved substantial platelet count recovery by the time of hospital discharge, even in cases with initial counts as low as ~3 × 10^9^/L. This favorable outcome is in line with the generally good prognosis reported for pediatric ITP [34,35,36]. It also corroborates earlier work demonstrating the efficacy of immunomodulatory treatments (like IVIG) in rapidly raising platelet counts in children [37]. In this context, the observed rise in platelet count at discharge should not be interpreted as a definitive indicator of recovery or therapeutic success. Pediatric ITP is frequently a self-limited but relapsing condition, and all currently available treatments are intended for transient symptomatic control rather than cure. Without long-term follow-up, we cannot determine if platelet counts stayed stable or returned to baseline after discharge. Additionally, the inclusion criteria limited the study to inpatients, thereby excluding children with milder forms of ITP who were managed as outpatients. This selection bias may have influenced both the clinical and laboratory profiles observed in our cohort. Although bleeding manifestations were mostly mild even among hospitalized children, we acknowledge that our findings cannot be generalized to the broader pediatric ITP population. Several conditions observed in our cohort—such as rhinosinusitis, obesity, acne, and dental caries—are commonly encountered in the general pediatric population and may not represent true secondary causes of ITP. Their presence is more likely coincidental than etiologic. In the present analysis, we have considered these as coexisting conditions rather than secondary forms of ITP. This approach is consistent with current international hematology guidelines, which define secondary ITP as being triggered by specific underlying disorders such as autoimmune disease, chronic infection, or certain medications. These concurrent conditions influenced our management approach. This regional perspective provides novel epidemiologic data on pediatric ITP in an underrepresented population and underscores the influence of environmental and healthcare disparities on disease manifestation. In accordance with the IWG consensus criteria, conditions such as herpetic stomatitis or acute upper respiratory infections were classified as coexisting rather than secondary causes of ITP. Secondary ITP is defined by the presence of a persistent, systemic disorder directly responsible for immune-mediated platelet destruction (like autoimmune diseases, chronic viral infections such as hepatitis C, HIV, or EBV). None of our patients fulfilled these criteria, and all serological tests for chronic viral infections were negative. Therefore, these transient, self-limited infections were considered concurrent findings, consistent with standard hematological definitions.

### 4.3. Integrated Clinical Management and Prognostic Insights in Pediatric ITP

Patients with significant infections received targeted antimicrobial therapy alongside IVIG (for instance, children with ITP and pneumonia were treated with appropriate antibiotics such as meropenem), and metabolic disturbances like hypocalcemia were corrected with supplements during treatment. These minor, subclinical decreases in calcium may reflect transient, reversible factors such as acute illness, decreased nutritional intake during hospitalization, or underlying vitamin D deficiency, which is relatively prevalent in Romanian children. The high prevalence of mild hypocalcemia in our cohort likely reflects a combination of selection bias, due to inclusion of only acutely hospitalized children, and the heightened clinical vigilance in our tertiary center, where extensive metabolic screening is routinely performed. It is important to note that certain conditions listed (such as hypocalcemia or hyponatremia) are pathophysiological syndromes or lab abnormalities rather than primary diseases. Most hypocalcemia cases in our cohort were mild (30 patients), with values only slightly below (0.2–0.4 mg/dL) the laboratory reference range and without clinical sequelae. According to the laboratory reference intervals in Romania (8.6–10.2 mg/dL), mild hypocalcemia was defined as serum calcium between 8.1–8.6 mg/dL. Moderate hypocalcemia, defined as serum calcium values between 7.6 and 8.0 mg/dL, was observed in 15 patients from our group. These transient, subclinical abnormalities are commonly encountered in acutely ill children, particularly in the context of intercurrent infections or decreased dietary intake. However, their presence still complicates the strict application of diagnostic criteria for ITP as a diagnosis of exclusion. Therefore, while these children were clinically managed as ITP, we acknowledge that unexplained laboratory abnormalities, even if mild, may limit the diagnostic certainty in some cases. Beyond its metabolic significance, calcium also serves critical roles in immunologic and hematologic function. Intracellular calcium flux is essential for T-cell receptor signaling, cytokine release, and platelet activation. Experimental studies have shown that calcium imbalance can modulate immune tolerance and megakaryocyte maturation. Therefore, transient hypocalcemia observed in our cohort may reflect broader immune activation associated with acute ITP rather than act as a primary pathogenic driver. Importantly, correction of serum calcium levels during hospitalization did not alter platelet recovery kinetics under standard first-line therapy (IVIG and corticosteroids), suggesting that hypocalcemia was an epiphenomenon rather than a determinant of therapeutic response. Nevertheless, future research incorporating vitamin D and parathyroid hormone measurements could further elucidate potential immunometabolic interactions influencing disease expression and recovery in pediatric ITP. An integrative approach combining demographic, metabolic, and hematologic parameters might facilitate early ITP recognition. Future studies could validate a ‘composite diagnostic score’ integrating serum calcium, infection markers, and platelet kinetics, potentially improving differentiation between transient and persistent forms. Addressing these underlying issues in parallel with ITP-specific therapy was deemed crucial for optimal recovery. This approach is supported by previous reports indicating that infections and metabolic disturbances frequently coexist with pediatric ITP [38] and can act as triggers for disease onset [39]. For example, viral illnesses have been shown to precipitate immune dysregulation, leading to ITP in susceptible children [40]. Our findings underscore the importance of a thorough initial workup to identify any precipitating or co-morbid conditions. Treating those conditions concurrently with standard ITP therapy likely contributed to the overall success in achieving platelet recovery, highlighting the need for an integrative management strategy in pediatric ITP. Despite the extensive clinical and laboratory evaluation at admission, we acknowledge that certain serious conditions may initially mimic ITP and only declare themselves over time. Disorders such as systemic lupus erythematosus, autoimmune thyroiditis, and even early acute leukemia can present with isolated thrombocytopenia at onset and may not be distinguishable from primary ITP during a brief hospital stay [33]. Without long-term follow-up, these cases remain diagnostically ambiguous. Previous studies have suggested that up to 10% of children initially thought to have ITP may ultimately receive an alternative diagnosis following extended observation [21]. As our study included only newly diagnosed cases during hospitalization, we recognize that a proportion of true secondary ITP cases or ITP mimics may have gone unrecognized. This represents a key limitation of our diagnostic certainty and underscores the importance of longitudinal evaluation in pediatric hematologic practice. Another limitation of this study is the absence of a standardized bleeding severity assessment tool. Although all bleeding manifestations (such as petechiae, ecchymoses, and mucosal hemorrhages) were systematically recorded, their severity was not graded using validated clinical scales such as the Buchanan bleeding score or the WHO bleeding scale. This omission limited our ability to establish quantitative correlations between bleeding severity, platelet count at presentation, and response to therapy. Incorporating such validated scoring systems in future studies would enable a more precise evaluation of hemorrhagic risk, facilitate inter-study comparability, and improve prognostic assessment in pediatric ITP. While clinical symptoms such as petechiae, ecchymoses, and mucosal bleeding were documented, the study did not employ a validated bleeding score, such as the Buchanan or WHO bleeding scale, to quantify the severity of hemorrhagic manifestations. As a result, we were unable to perform a structured correlation between bleeding severity and platelet count at presentation. Future studies should incorporate formal bleeding assessment tools to enable more clinically meaningful stratification of ITP severity and better inform therapeutic decision-making. Finally, our study provides a broader clinical perspective by comparing pediatric ITP characteristics to known adult patterns. Although splenectomy remains a definitive therapeutic option in selected refractory cases, the absence of such interventions in our cohort reflects contemporary pediatric practice trends favoring non-surgical management. Prior investigations in adults have reported a higher tendency toward chronic, refractory ITP and a more protracted disease course [35]. In contrast, pediatric ITP, consistent with our cohort outcomes, appears more often to be an acute, self-limited condition with a strong propensity for spontaneous or therapy-induced remission. Many children in our study, despite presenting with markedly low platelet counts, showed near-complete platelet count restoration by discharge. This observation supports the concept that children have a robust bone marrow regenerative capacity and immune recovery potential compared to adults [36,37]. It also suggests that fundamental age-related differences in immune system dynamics influence the disease trajectory. That said, pediatric ITP is heterogeneous, and a small subset of patients may experience chronic or recurrent courses. Identifying which children are at risk for a protracted illness remains an important challenge. Some researchers have begun examining clinical predictors present at diagnosis that might forecast disease persistence or refractoriness [18]. Similarly, recent analyses emphasize the need for early markers of refractory disease to guide personalized interventions [41,42,43,44]. Another intriguing finding in our cohort was the difference in platelet recovery between boys and girls, raising the possibility that sex-specific factors, genetic or hormonal, could modulate the immune response in ITP [42]. This facet merits further investigation in future studies. In summary, our three-year observational study of pediatric ITP reinforces known favorable outcomes and epidemiological patterns in children, while also shedding light on the nuances of disease presentation and recovery. These insights contribute to a more coherent understanding of pediatric ITP and may inform more tailored management strategies moving forward.

To our knowledge, this is one of the first comprehensive observational studies of hospitalized children with newly diagnosed ITP conducted in Eastern Europe, focusing not only on acute clinical presentation and platelet response to standardized first-line therapy, but also on the prevalence and characterization of coexisting clinical conditions. The identification of a relatively low proportion of truly isolated primary ITP (15%), alongside a high prevalence of mild, often asymptomatic laboratory and clinical findings, provides new insight into the heterogeneity of ITP presentations in hospitalized pediatric patients. The implications of our findings are twofold. First, they underscore the importance of thorough, multidisciplinary evaluation for potential coexisting conditions in children diagnosed with ITP, even when initial presentation appears typical. Second, our results highlight the need for tailored follow-up strategies and diagnostic vigilance, particularly in settings where coexisting clinical conditions or mild laboratory abnormalities are prevalent. For clinical practice, this may translate into more cautious application of the “diagnosis of exclusion” paradigm and more individualized management plans.

Given the lack of post-discharge follow-up and standardized bleeding severity assessment, our results cannot be extrapolated to the broader pediatric ITP population or used to infer long-term outcomes. The observed coexisting clinical conditions likely reflect incidental coexistence rather than secondary causes of ITP. Moreover, the inpatient-only design may have introduced selection bias toward more clinically severe cases. Future prospective studies that include outpatient follow-up, validated bleeding scores, and stratified treatment protocols are needed to deepen our understanding of disease course, optimize individualized management, and improve outcome prediction in pediatric ITP.

## 5. Conclusions

This retrospective, hospital-based study provides a descriptive overview of clinical characteristics, coexisting clinical conditions, and short-term hematologic response in children diagnosed with newly onset ITP during hospitalization. Most patients demonstrated an increase in platelet counts during admission, but this finding should be interpreted as an acute response to first-line interventions rather than a definitive therapeutic success.

## Figures and Tables

**Table 1:** Patient Characteristics of the Pediatric ITP patients (N = 100).

Characteristic	Subcategory	Patients Number
Age group (years)	0–5	40
	6–10	20
	11–15	25
	>15	15
Sex	Male	70
	Female	30
Residential background	Urban	55
	Rural	45

**Table 2:** Distribution of petechiae and ecchymoses by anatomical localization and gender. Percentages were calculated separately for female and male patients, using the total number of patients within each sex as the denominator (*n* = 30 females; *n* = 70 males).

Localization	Petechiae		Ecchymoses	
Sex Distributions	F (Number of Patients/Percentages (%))	M (Number of Patients/Percentages (%))	F (Number of Patients/Percentages (%))	M (Number of Patients/Percentages (%))
Lower limbs	5 (16.66%)	60 (85.71%)	20 (66.66%)	40 (57.14%)
Abdomen	10 (33.33%)	50 (71.42%)	10 (33.33%)	30 (42.85%)
Thorax	15 (50.00%)	25 (35.29%)	5 (16.66%)	25 (35.71%)
Upper limbs	5 (16.66%)	29 (41.17%)	10 (33.33%)	20 (28.57%)
Face	15 (50.00%)	4 (5.88%)	10 (33.33%)	15 (21.42%)
Neck	5 (16.66%)	12 (17.64%)	5 (16.66%)	5 (7.14%)
The hard palate	5 (16.66%)	12 (17.64%)	-	-
Mouth	10 (33.30%)	4 (5.88%)	0 (0.00%)	10 (14.28%)
Venous access	-	-	0 (0.00%)	10 (14.28%)
Generalized	0 (0.00%)	4 (5.88%)	-	-
Lower back	-	-	5 (16.66%)	5 (7.14%)

**Table 3:** Therapeutic management of ITP in the study cohort.

Therapeutic Category	Medication	Route of Administration	Pediatric Dosage	Indication
First-line therapy	IVIG	Intravenous infusion over 4–6 h	0.8–1 g/kg/day for 1–2 consecutive days	First-line treatment for newly diagnosed ITP, severe thrombocytopenia, or mucocutaneous bleeding; rapid platelet response within 24–48 h.
	Prednisone	Oral	1–2 mg/kg/day for 5–7 days	Used when IVIG was contraindicated, unavailable, or in mild, non-life-threatening bleeding.
Second-line/rescue therapy	Platelet transfusion	Intravenous (slow infusion)	5–10 mL/kg (single donor unit)	Reserved for active bleeding or platelet count <10 × 10^9^/L, according to institutional protocols.
Supportive/adjunctive therapy	Desloratadine	Oral	Age-adjusted standard dosing	Prescribed for allergic symptom control.
	Calcium gluconate	Oral/Intravenous	50–100 mg/kg/day (elemental calcium equivalent)	Correction of mild/moderate hypocalcemia frequently observed in ITP patients.
	Omeprazole	Oral	1 mg/kg/day	Gastroprotection during corticosteroid therapy.
	Rutoside	Oral	Per manufacturer recommendations	Supportive nutritional therapy.
	Cefoperazone–sulbactam/Meropenem	Intravenous	Standard antimicrobial pediatric doses	Used to treat bacterial infections when clinically indicated.
	Acetaminophen	Oral	10–15 mg/kg every 6–8 h as needed	Symptomatic antipyretic/analgesic.
	Vitamin C	Oral	Standard pediatric dosing	Antioxidant and supportive therapy.

**Table 4:** Classification of Isolated ITP Cases and ITP with Coexisting Clinical Conditions by Gender.

Diagnosis	Total Number of Patients	Number of Male Patients	Number of Female Patients
Thrombocytopenic Purpura (All Patients)	100	70	30
Isolated ITP (No coexisting clinical conditions)	15	15	0
ITP with coexisting Clinical Conditions			
Thrombocytopenic Purpura + Mild/Moderate Hypocalcemia	45	25	20
Thrombocytopenic Purpura + Herpetic Stomatitis	20	20	0
Thrombocytopenic Purpura + Rhinosinusitis/Adenoiditis	20	20	0
Thrombocytopenic Purpura + Obesity	20	15	5
Thrombocytopenic Purpura + Acute Pneumonia	15	5	10
Thrombocytopenic Purpura + Epistaxis	15	15	0
Thrombocytopenic Purpura + Juvenile Acne	15	10	5
Thrombocytopenic Purpura + Iron Deficiency Anemia	10	5	5
Thrombocytopenic Purpura + Dental Caries	10	5	5
Thrombocytopenic Purpura + Serous Otitis	10	10	0
Thrombocytopenic Purpura + Underweight	10	5	5
Thrombocytopenic Purpura + Hyponatremia	5	5	0
Thrombocytopenic Purpura + Acute Tracheobronchitis	5	0	5
Thrombocytopenic Purpura + Eosinophilia	5	0	5
Thrombocytopenic Purpura + Tetany	5	0	5
Thrombocytopenic Purpura + Epilepsy	5	5	0
Thrombocytopenic Purpura + Maxillary Sinusitis	5	5	0

## Data Availability

Data are contained within the article.

## References

[1] Rodeghiero F., Stasi R., Gernsheimer T., Michel M., Provan D., Arnold D.M., Bussel J.B., Cines D.B., Chong B.H., Cooper N. (2009). Standardization of terminology, definitions and outcome criteria in immune thrombocytopenic purpura of adults and children: Report from an international working group. Blood.

[2] Buchanan G.R. (2005). Thrombocytopenia during childhood: What the pediatrician needs to know. Pediatr. Rev..

[3] Neunert C., Lim W., Crowther M., Cohen A., Solberg L., Crowther M.A. (2011). The American Society of Hematology 2011 evidence-based practice guideline for immune thrombocytopenia. Blood.

[4] Lev P.R., Goette N.P., Marta R.F. (2020). Pathophysiological mechanisms leading to low platelet count in immune thrombocyto penia. J. Immunological. Sci..

[5] Zheng S.S., Perdomo J.S. (2024). Desialylation and Apoptosis in Immune Thrombocytopenia: Implications for Pathogenesis and Treatment. Curr. Issues Mol. Biol..

[6] Cines D.B., Cuker A., Semple J.W. (2014). Pathogenesis of immune thrombocytopenia. Presse Med..

[7] Giordano P., Lassandro G., Barone A., Cesaro S., Fotzi I., Giona F., Gorio C., Maggio A., Miano M., Marzollo A. (2023). Long-Term Use of Eltrombopag in Children with Chronic Immune Thrombocytopenia: Extended Real-Life Retrospective Multicenter Experience of the Italian Association of Pediatric Hematology and Oncology. Front. Med..

[8] Giordano P., Lassandro G., Barone A., Cesaro S., Fotzi I., Giona F., Ladogana S., Miano M., Marzollo A., Nardi M. (2020). Use of Eltrombopag in Children with Chronic Immune Thrombocytopenia (ITP): A Real-Life Retrospective Multicenter Experience of the Italian Association of Pediatric Hematology and Oncology (AIEOP). Front. Med..

[9] Matzdorff A., Alesci S.R., Gebhart J., Holzhauer S., Hütter-Krönke M.L., Kühne T., Meyer O., Ostermann H., Pabinger I., Rummel M. (2023). Expert Report on Immune Thrombocytopenia: Current Diagnostics and Treatment—Recommendations from an Expert Group from Austria, Germany, and Switzerland. Oncol. Res. Treat..

[10] Najaoui A., Bakchoul T., Stoy J., Bein G., Rummel M.J., Santoso S., Sachs U.J. (2012). Autoantibody-mediated complement activation on platelets is a common finding in patients with immune thrombocytopenic purpura (ITP). Eur. J. Haematol..

[11] Sakakura M., Wada H., Tawara I., Nobori T., Sugiyama T., Sagawa N., Shiku H. (2007). Reduced Cd41Cd251 T cells in patients with idiopathic thrombocytopenic purpura. Thromb. Res..

[12] Li X., Zhong H., Bao W., Boulad N., Evangelista J., Haider M.A., Bussel J., Yazdanbakhsh K. (2012). Defective regulatory B-cell compartment in patients with immune thrombocytopenia. Blood.

[13] Li S., Wang L., Zhao C., Li L., Peng J., Hou M. (2007). CD8+ T cells suppress autologous megakaryocyte apoptosis in idiopathic thrombocytopenic purpura. Br. J. Haematol..

[14] Vrbensky J.R., Nazy I., Toltl L.J., Ross C., Ivetic N., Smith J.W., Kelton J.G., Arnold D.M. (2018). Megakaryocyte apoptosis in immune thrombocytopenia. Platelets.

[15] Olsson B., Andersson P.O., Jernås M., Jacobsson S., Carlsson B., Carlsson L.M.S., Wadenvik H. (2003). T-cell-mediated cytotoxicity toward platelets in chronic idiopathic thrombocytopenic purpura. Nat. Med..

[16] (2022). Medical laboratories—Requirements for Quality and Competence.

[17] Provan D., Stasi R., Newland A.C., Blanchette V.S., Bolton-Maggs P., Bussel J.B., Chong B.H., Cines D.B., Gernsheimer T.B., Godeau B. (2010). International consensus report on the investigation and management of primary immune thrombocytopenia. Blood.

[18] Neunert C.E., Cooper N. (2018). Evidence-Based Management of Immune Thrombocytopenia: ASH Guideline Update. Hematol. Am. Soc. Hematol. Educ. Program..

[19] Brîndușe L.A., Eclemea I., Neculau A.E., Păunescu B.A., Bratu E.C., Cucu M.A. (2024). Rural versus urban healthcare through the lens of health behaviors and access to primary care: A post-hoc analysis of the Romanian health evaluation survey. BMC Health Serv. Res..

[20] Shaw J., Kilpatrick K., Eisen M., Tarantino M. (2020). The incidence and clinical burden of immune thrombocytopenia in pediatric patients in the United States. Platelets.

[21] Consolini R., Costagliola G., Spatafora D. (2017). The Centenary of Immune Thrombocytopenia—Part 2: Revising Diagnostic and Therapeutic Approach. Front. Pediatr..

[22] Cuker A., Neunert C.E. (2016). How I treat refractory immune thrombocytopenia. Blood.

[23] Cole C. (2017). Lessons in the diagnosis and management of immune thrombocytopenic purpura in children. J. Paediatr. Child Health.

[24] Grace R.F., Neunert C. (2016). Second-line therapies in immune thrombocytopenia. Hematol. Am. Soc. Hematol. Educ. Program..

[25] Adewoyin A.S., Nwogoh B. (2014). Peripheral blood film—A review. Ann. Ib. Postgrad. Med..

[26] Frederiksen H., Schmidt K. (1999). The incidence of idiopathic thrombocytopenic purpura in adults increases with age. Blood.

[27] Grimaldi-Bensouda L., Nordon C., Michel M., Viallard J.-F., Adoue D., Magy Bertrand N., Durand J.-M., Quittet P., Fain O., Bonnotte B. (2016). Immune thrombocytopenia in adults: A prospective cohort study of clinical features and predictors of outcome. Haematologica.

[28] Fischer A., Provot J., Jais J.P., Alcais A., Mahlaoui N., Members of the CEREDIH French PID Study Group (2017). Autoimmune and inflammatory manifestations occur frequently in patients with primary immunodeficiencies. J. Allergy Clin. Immunol..

[29] Hadjadj J., Aladjidi N., Fernandes H., Leverger G., Magérus-Chatinet A., Mazerolles F., Stolzenberg M.-C., Jacques S., Picard C., Members of the French Reference Center for Pediatric Autoimmune Cytopenia (2019). Pediatric Evans syndrome is associated with a high frequency of potentially damaging variants in immune genes. Blood.

[30] Mithoowani S., Cervi A., Shah N., Ejaz R., Sirotich E., Barty R., Li N., Nazy I., Arnold D.M. (2020). Management of major bleeds in patients with immune thrombocytopenia. J. Thromb. Haemost..

[31] Kohli R., Chaturvedi S. (2019). Epidemiology and clinical manifestations of immune thrombocytopenia. Hamostaseologie.

[32] Peng J., Friese P., Heilmann E., George J.N., Burstein S.A., Dale G.L. (1994). Aged platelets have an impaired response to thrombin as quantitated by P-selectin expression. Blood.

[33] Neunert C., Terrell D.R., Arnold D.M., Buchanan G., Cines D.B., Cooper N., Cuker A., Despotovic J.M., George J.N., Grace R.F. (2019). American Society of Hematology 2019 guidelines for immune thrombocytopenia. Blood Adv..

[34] Bruin M., Bierings M., Uiterwaal C., Évész T.R., Bode L., Wiesman M.-E., Kuijpers T.W., Tamminga R., De Haas M. (2004). Platelet count, previous infection and FCGR2B genotype predict development of chronic disease in newly diagnosed idiopathic thrombocytopenia in childhood: Results of a prospective study. Br. J. Haematol..

[35] Heitink-Pollé K.M., Nijsten J., Boonacker C.W., De Haas M., Bruin M.C.A. (2014). Clinical and laboratory predictors of chronic immune thrombocytopenia in children: A systematic review and meta-analysis. Blood.

[36] Tamminga R., Berchtold W., Bruin M., Buchanan G.R., Kuehne T. (2009). Possible lower rate of chronic ITP after IVIG for acute childhood ITP: An analysis from registry I of the Intercontinental Cooperative ITP Study Group (ICIS). Br. J. Haematol..

[37] Arnold D.M., Crowther M.A., Cook R.J., Sigouin C., Heddle N.M., Molnar L., Cook D.J. (2006). Utilization of platelet transfusions in the intensive care unit: Indications, transfusion triggers, and platelet count responses. Transfusion.

[38] Rao M.P., Boralessa H., Morgan C., Soni N., Goldhill D.R., Brett S.J., Contreras M. (2002). Blood component use in critically ill patients. Anaesthesia.

[39] van Baarle F.L., van de Weerdt E.K., van der Velden W.J., Ruiterkamp R.A., Tuinman P.R., Ypma P.F., Bergh W.M.v.D., Demandt A.M., Kerver E.D., Jansen A.G. (2023). Platelet transfusion before CVC placement in patients with thrombocytopenia. N. Engl. J. Med..

[40] Grimaldi-Bensouda L., Nordon C., Leblanc T., Abenhaim L., Allali S., Armari-Alla C., Berger C., Courcoux M.-F., Fouyssac F., Guillaumat C. (2017). Childhood immune thrombocytopenia: A nationwide cohort study on condition management and outcomes. Pediatr. Blood Cancer.

[41] Kime C., Klima J., Rose M.J., O’Brien S.H. (2013). Patterns of inpatient care for newly diagnosed immune thrombocytopenia in US children’s hospitals. Pediatrics.

[42] Jubelirer S.J., Harpold R. (2002). The role of the bone marrow examination in the diagnosis of immune thrombocytopenic purpura: Case series and literature review. Clin. Appl. Thromb. Hemost..

[43] Allegra A., Cicero N., Mirabile G., Giorgianni C.M., Gangemi S. (2023). Novel Biomarkers for Diagnosis and Monitoring of Immune Thrombocytopenia. Int. J. Mol. Sci..

[44] Semple J.W., Rebetz J., Maouia A., Kapur R. (2020). An update on the pathophysiology of immune thrombocytopenia. Curr. Opin. Hematol..

